# Chemometric analysis of biofluids from mice experimentally infected with *Schistosoma mansoni*

**DOI:** 10.1186/1756-3305-4-179

**Published:** 2011-09-19

**Authors:** Jia V Li, Jasmina Saric, Yulan Wang, Jennifer Keiser, Jürg Utzinger, Elaine Holmes

**Affiliations:** 1 Section of Biomolecular Medicine, Department of Surgery and Cancer, Faculty of Medicine, Imperial College London, London, UK; 2 State Key Laboratory of Magnetic Resonance and Atomic and Molecular Physics, Wuhan Centre for Magnetic Resonance, Wuhan Institute of Physics and Mathematics, Chinese Academy of Sciences, Wuhan, People's Republic of China; 3 Department of Medical Parasitology and Infection Biology, Swiss Tropical and Public Health Institute, Basel, Switzerland; 4 University of Basel, Basel, Switzerland; 5 Department of Epidemiology and Public Health, Swiss Tropical and Public Health Institute, Basel, Switzerland

## Abstract

**Background:**

The urinary metabolic fingerprint of a patent *Schistosoma mansoni *infection in the mouse has been characterized using spectroscopic methods. However, the temporal dynamics of metabolic alterations have not been studied at the systems level. Here, we investigated the systems metabolic changes in the mouse upon *S. mansoni *infection by modeling the sequence of metabolic events in urine, plasma and faecal water.

**Methods:**

Ten female NMRI mice, aged 5 weeks, were infected with 80 *S. mansoni *cercariae each. Ten age- and sex-matched mice remained uninfected and served as a control group. Urine, plasma and faecal samples were collected 1 day before, and on eight time points until day 73 post-infection. Biofluid samples were subjected to ^1^H nuclear magnetic resonance (NMR) spectroscopy and multivariate statistical analyses.

**Results:**

Differences between *S. mansoni*-infected and uninfected control mice were found from day 41 onwards. One of the key metabolic signatures in urine and faecal extracts was an alteration in several gut bacteria-related metabolites, whereas the plasma reflected *S. mansoni *infection by changes in metabolites related to energy homeostasis, such as relatively higher levels of lipids and decreased levels of glucose. We identified 12 urinary biomarkers of *S. mansoni *infection, among which hippurate, phenylacetylglycine (PAG) and 2-oxoadipate were particularly robust with regard to disease progression. Thirteen plasma metabolites were found to differentiate infected from control mice, with the lipid components, D-3-hydroxybutyrate and glycerophosphorylcholine showing greatest consistency. Faecal extracts were highly variable in chemical composition and therefore only five metabolites were found discriminatory of infected mice, of which 5-aminovalerate was the most stable and showed a positive correlation with urinary PAG.

**Conclusions:**

The composite metabolic signature of *S. mansoni *in the mouse derived from perturbations in urinary, faecal and plasma composition showed a coherent response in altered energy metabolism and in gut microbial activity. Our findings provide new mechanistic insight into host-parasite interactions across different compartments and identified a set of temporally robust biomarkers of *S. mansoni *infection, which might assist in derivation of diagnostic assays or metrics for monitoring therapeutic response.

## Background

Schistosomiasis is the most important water-based disease worldwide, and belongs to the neglected tropical diseases. The causative agents are blood flukes of the genus *Schistosoma*. Six species parasitize humans, of which *Schistosoma haematobium*, *S. mansoni *and *S. japonicum *exhibit the widest geographical distribution, public health burden and socioeconomic impact [1-3]. Close to 800 million individuals are at risk of schistosomiasis, more than 200 million are infected and the global burden due to schistosomiasis might be as high as 4.5 million disability-adjusted life years (DALYs) [2,4].

Given the extend of the schistosomiasis problem, and the fact that treatment relies on a single drug, praziquantel, which raises issues with respect to resistance, there is an unmet need for acquiring a deeper understanding of the bidirectional communication between the parasite and the mammalian host with a view to identifying new methods of control and potential drug targets. One potential approach to investigating the developing relationship between the parasite and its host is metabolic profiling. Several studies have shown that metabolic profiling methods such as ^1^H nuclear magnetic resonance (NMR) spectroscopy, ultra-performance liquid chromatography (UPLC)-mass spectrometry (MS) and capillary electrophoresis (CE)-MS, coupled with multivariate data analyses hold promise for probing the metabolic consequences of parasitic infections, including schistosomiasis in both murine and human hosts [5-8]. Murine models experimentally infected with either *S. mansoni *or *S. japonicum *showed stimulated glycolysis (i.e. increased levels of pyruvate), and perturbation of the tricarboxylic acid (TCA) cycle (i.e. decreased concentrations of citrate, succinate, and 2-oxoglutarate). Alterations in microbiota-related metabolites (e.g. acetate, butyrate, propionate, hippurate, *p*-cresol glucuronide and phenylacetylglycine) have also been observed indicating cross-talk between schistosomes, the murine host and its enteric gut microbiota [6,9,10]. Some of these findings were replicated in a recent human cohort study on *S. mansoni*-infected individuals in Uganda [5]. Metabonomic investigation of urinary profiles has also shown infection-related effects in a range of molecular markers related to energy metabolism and liver function [5]. In addition to prospecting for biomarkers of infection in readily accessible biofluids such as urine and faeces, metabolic studies have focused on integrating biochemical signatures across multiple biological samples in a move towards a systems biology approach. This systems-wide application of metabolic profiling has been adopted in a diversity of fields, such as pharmaceutical research and development, nutrition, toxicology and systems biology, among others [11-13]. Specific application of metabolic profiling in parasitic infection biology has been reviewed up to date [7].

In the current study, we expand the metabolic profiling approach to characterize the dynamic and integrated response of the murine host to a *S. mansoni *infection during acute and chronic stages in urine, plasma and faecal extracts over a 73-day period in order to broaden the coverage of the metabolome and to ascertain which of the candidate biomarkers hold promise in terms of early detection, robustness and sensitivity. Moreover, profiling of the faecal metabolome may aid in the interpretation of the reported perturbation in gut microbial metabolism, thus far expressed *via *changes in urinary excretion profiles. Insight gained at the systems metabolic level will deepen the understanding of the mechanisms of infection and disease, and might give rise to novel diagnostic and prognostic markers of infection or response to treatment.

## Methods

### Animal model

Details of the *S. mansoni*-mouse model employed in the current investigation have been described elsewhere [14]. In brief, 20 NMRI female mice, aged 3 weeks, were purchased from RCC (Füllinsdorf, Switzerland) and group-housed (five animals per cage). Mice had free access to food pellets (PAB45-NAFAG 9009, Provimi Kliba; Kaiseraugst, Switzerland) and community tap water throughout the experiment. Mice were kept under stable environmental conditions (temperature, ~22°C; humidity, ~70%; day/night cycle, 12/12 hours). We adhered to laws of the relevant regional and national authorities for animal handling, infection, sample collection, euthanizing and dissection (permission no. 2081).

After an acclimatization period of 2 weeks to avoid confounding stress-related metabolic activity, half of the mice (*n *= 10) were infected subcutaneously with 80 *S. mansoni *cercariae each. The remaining 10 mice were left uninfected, and hence served as control group. Mice were approximately 5 weeks old at the designated day of infection (day 0) and had a mean weight of 22.9 g (range: 19.7-24.9 g). One mouse in the control group became moribund early on in the experiment, and was hence excluded from all further analyses.

### Sample collection

Biofluids were collected between 08:00 and 11:00 hours in order to minimize diurnal variation in biofluid compositions. At least 30 μl of urine and two faecal pellets were collected from each mouse directly into Petri dishes by gently rubbing their abdomen. Mice failing to let sufficient quantities of either urine or faeces were placed individually into sterile empty plastic cages and observed until sufficiently large quantities of urine and/or faecal pellets were generated. Urine and faecal samples were collected into individually labelled Eppendorf tubes, and immediately transferred onto dry ice prior to storage at -40°C.

Approximately 45 μl of blood was collected *via *tail snip into a haematocrit capillary coated with sodium heparin. Capillaries were centrifuged at 4,000 *g *for 5 min and the packed cell volume (PCV) was recorded. Plasma samples were transferred into separate Eppendorf tubes, transferred immediately onto dry ice and subsequently stored at -40°C.

Sampling of urine, faecal pellets and blood was carried out nine times over the course of the study, namely 1 day before infection (D_-1_) and on days 13, 27, 41, 48, 53, 61, 67 and 73 post-infection, in order to capture the overt infection as well as early infection stages. Sampling of non-infected control mice took place at the same time points.

### NMR spectroscopy

A total of 30 μl urine of each sample was mixed with 25 μl of 0.2 M sodium phosphate buffer (pH = 7.4, 0.01% sodium 3-(trimethylsilyl) propionate-2,2,3,3-d_4 _(TSP), 3 mM Na_3_N) and transferred into a 1.7-mm outer diameter microtube. Two faecal pellets from each mouse at each time point were manually homogenized in 700 μl of the same phosphate buffer in 2-ml Eppendorf tubes. Homogenates were sonicated at room temperature for 30 min in order to fully dissolve the water-soluble components and also reduce the effect of micro-organisms on the samples. The centrifugation was carried out at 11,000 *g *for 10 min and 550 μl of supernatant was transferred into a 5-mm outer diameter glass tube pending NMR analysis. A total of 25 μl of plasma for each sample was mixed with 25 μl of saline (D_2_O:H_2_O = 9:1, v:v, 0.9% (w/v) NaCl), and subsequently transferred into 1.7-mm outer diameter microtubes.

^1^H NMR spectra were acquired using a Bruker DRX 600 MHz spectrometer (Rheinstetten, Germany) with a 5-mm TXI probe operating at 600.13 MHz. The field was locked to the ^2^H resonance of the D_2_O solvent. In order to suppress the large water signal, a standard one-dimensional (1-D) NMR pulse [recycle delay (RD)-90°-*t_1_*-90°-*t_m_*-90°-acquire free induction decay (FID)] was employed for the acquisition of all spectra. The water peak was suppressed by selective irradiation during RD of 2 s and mixing time (*t_m_*) of 100 ms and *t_1_*was fixed to 3 μs. The 90° pulse length was adjusted to ~10 μs. A total of 256 scans were recorded into 32 k data points with a spectral width of 20 ppm. An exponential function was applied to the FID prior to the Fourier transformation, which induced line broadening by 0.3 Hz [15]. In order to account for the smaller molecular components, a Carr-Purcell-Meiboom-Gill (CPMG) pulse sequence was applied additionally. Both ^1^H standard NMR and CPMG spectra were obtained using the same parameters except that the CPMG spectra were acquired using a spin-echo pulse sequence: [RD-90°-(τ-180°-τ)_n_-acquire FID]. Using this pulse sequence allows visualization of the low molecular weight metabolites whilst retaining some residual resonances from the more mobile lipid moieties [16]. For the CPMG experiment, a spin relaxation delay (2nτ) of 200 ms was used [15]. Diffusion-edited ^1^H NMR spectra with water suppression were acquired for selected plasma samples using the bipolar-pair-longitudinal-eddy-current pulse sequence. A total of 256 scans were recorded into 16 k data points with a spectral width of 20 ppm. This experiment was applied to investigate large molecules present in blood plasma (e.g. lipids) [15]. Due to NMR time constraints this type of the experiment was only applied to samples from selected time points.

Additionally, two-dimensional (2D) NMR experiments (^1^H-^1^H correlation spectroscopy [COSY] and ^1^H-^1^H total correlation spectroscopy [TOCSY]) [15], to assist metabolite identification, were carried out with selected urine samples. A total of 128 increments with 80 scans were accumulated into 2 k data points with a spectral width of 10 ppm for each dimension. The ^1^H-^1^H TOCSY NMR spectra were acquired by using the MLEV-17 sequence for the spin-lock with a spin-lock power of 6 kHz.

### Data reduction and multivariate data analyses

Spectra were pre-processed consisting of phase and baseline correction, calibration, digitization, removal of redundant spectral regions and normalization. For urine and faecal water, phase and baseline correction was achieved using an in-house developed MATLAB script (Dr. T. Ebbels, Imperial College London) and were referenced to the TSP resonance at δ ^1^H 0.00. Spectral regions corresponding to δ ^1^H 0-10.0 were digitized into 20,000 data points with the segment width of 0.0005 ppm and imported into MATLAB using an in-house developed MATLAB script (Dr. O. Cloarec, Imperial College London). Plasma spectra were processed similarly except that baseline correction and phasing were performed manually, whereas the anomeric proton from α-glucose at δ ^1^H 5.22 was used for calibration. The following spectral regions were removed to eliminate differences in the efficiency of water suppression, background noise or artefacts: for urine δ ^1^H 0-0.35, δ ^1^H 3.34-3.38, δ ^1^H 4.60-5.05, δ ^1^H 9.6-10.0 and δ ^1^H 5.16-6.35; for faecal water δ ^1^H 4.7-4.9, δ ^1^H 0-0.3 and δ ^1^H 9.1-10.0; for plasma δ ^1^H 3.33-3.36, δ ^1^H 4.40-5.15, δ ^1^H 0-0.7 and δ ^1^H 5.4-10.0. Subsequently normalization to total spectral area was carried out prior to employing multivariate data analysis approaches, including principal component analysis (PCA) and orthogonal-partial least square-discriminant analysis (O-PLS-DA) using criteria described elsewhere [17]. The number of components included in each model was determined using a cut-off when Q^2^*Y *fell below a 5% increase in cumulative predictive variance explained by the model. Metabolite assignments were made by use of 2D NMR pulse sequences, in-house chemical shift databases [9] or spiking of chemical standards.

Two urinary metabolites, i.e. hippurate and phenylacetylglycine (PAG), were found to be most highly correlated with the *S. mansoni *infection. Since these metabolites reflect gut microbial activity, their relationship with the faecal water profile was ascertained, using O-PLS regression analysis employing pre-processed faecal extract spectral data designated as ***X***matrix and the integrated hippurate resonance (δ ^1^H 7.815-7.858) and the integrated PAG resonance (δ ^1^H 7.400-7.446), respectively as the ***Y***matrix.

## Results

### Physiological observation of *S. mansoni *infection in mice

**Figure 1:**
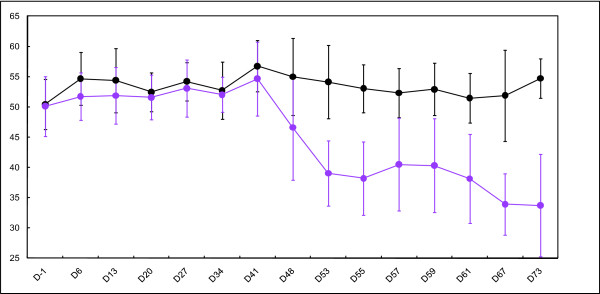
**Graph showing packed cell volume (PCV) ratio of uninfected control and *S. mansoni*-infected groups of mice across all the experimental time points**. The graph shows a significant decrease in PCV of infected mice from day 48 post-infection onwards. The error bars represent 2x standard deviation.

While the mean PCV of the control group remained constant over the course of the experiment, the mean PCV of *S. mansoni*-infected mice showed constant levels up to day 41 post-infection (PCV = 54.6%) and a slight reduction on 48 days post-infection (PCV = 46.6%). A steep decline occurred thereafter to a level as low as 33.7% at the end of the experiment, 73 days post-infection (Figure 1).

The mean *S. mansoni *worm burden in mice upon dissection 73 days post-infection was 36 worms with a standard deviation of 7 worms. No significant difference in mean body weight of *S. mansoni*-infected and non-infected control mice was observed over the course of the experiment.

### Metabolic profiling and multivariate data analysis on mouse urine

**Figure 2:**
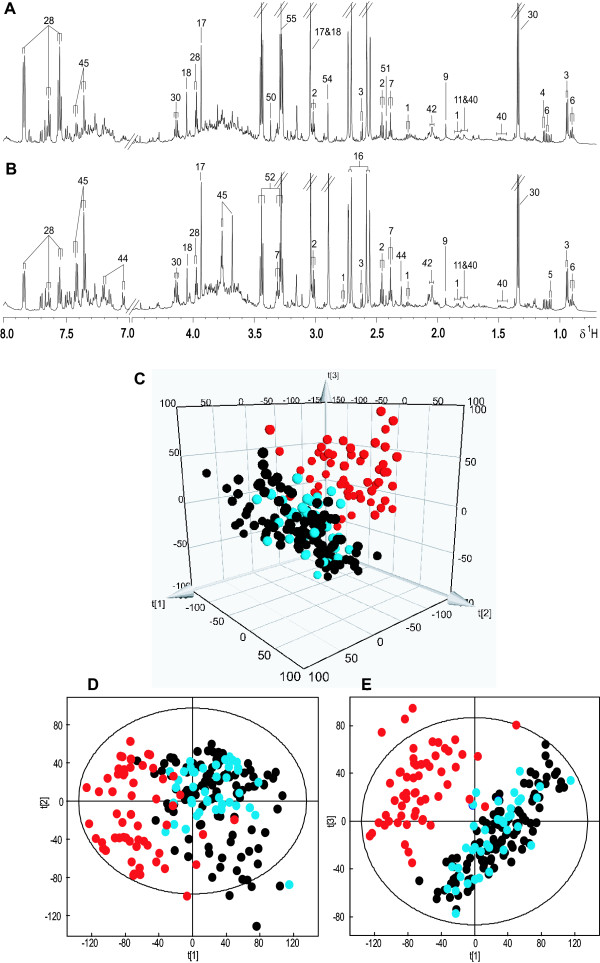
**Typical 600 MHz ^1^H NMR spectra of urine obtained from an uninfected (A) and a *S. mansoni*-infected (B) mouse at day 53 post-infection**. A 3-D PCA trajectory scores plot (C) derived from urinary spectra of both control (black), *S. mansoni*-infected mice at early stage (13-41 days post-infection, blue) and these infected mice at the late stage (48-73 days post-infection, red). D and E represent 2-D PCA scores plots constructed from first and second PCs (D), and first and third PCs (E), respectively.

Two representative ^1^H NMR spectra of urine, one obtained from an uninfected control mouse and the second one from a *S. mansoni*-infected mouse 53 days post-infection, are shown in Figures 2A and 2B. Lactate, lysine, citrate, 2-oxoglutarate, succinate, 3-ureidopropionic acid, acetate, trimethylamine, creatine, creatinine, taurine, trimethylamine *N*-oxide (TMAO), alanine, arginine, hippurate, glycine, 2-oxoadipate, 2-oxoisocaproate, 3-methyl-2-oxovalerate, PAG and 2-oxoisovalerate were identified in the urinary metabolic spectra of the control mice, whereas *p*-cresol glucuronide was only detected in urine of *S. mansoni*-infected mice from day 48 post-infection onwards. Chemical shifts and multiplicities of signals are summarized in Table 1.

**Table 1:** Identified metabolites in ^1^H NMR spectra of urine (u), plasma (p) and faecal water (f) obtained from mice with or without an infection of *S.mansoni*, together with the respective chemical shifts and signal multiplicities

No	Metabolites	H group	δ ^1^H (multiplicity*)	Biofluids
1	2-oxoadipate	β-CH_2_; γ-CH_2_; δ-CH_2_	2.77(t); 1.82(m); 2.22(t)	u
2	2-oxoglutarate	β-CH_2_; γ-CH_2_	3.01(t); 2.45(t)	u
3	2-oxoisocaproate	β-CH_2_; γ-CH; 2x CH_3_	2.61(d); 2.1(m); 0.94(d)	u
4	2-oxoisovalerate	β-CH; 2x CH_3_	3.02(m); 1.13(d)	u/f
5	3-carboxy-2-methyl-3-oxopropanamine	β-CH; CH_3_; γ-CH_2_	2.49(m); 1.08(d); 3.19(m), 3.56(m), 3.72(m)	u
6	3-methyl-2-oxovalerate	β-CH; β-CH_3_; γ-CH_2_(i); γ-CH_2_(ii); γ-CH_3_	2.93(m); 1.1(d); 1.7(m); 1.46(m); 0.9(t)	u/f
7	3-ureidopropionic acid	α-CH_2_; β-CH_2_	2.38(t); 3.31(t)	u
8	5-aminovalerate	α-CH_2_; β-CH_2_; γ-CH_2_; δ-CH_2_	2.24(t); 1.64(m); 1.66(m); 3.02(t)	f
9	acetate	CH_3_	1.93(s)	u/p/f
10	alanine	CH; CH_3_	3.77(q); 1.47(d)	p/f
11	arginine	α-CH; β-CH_2_; γ-CH_2_; δ-CH_2_	3.78(t); 1.92(m); 1.65(m); 3.20(t)	u/f
12	asparagine	α-CH; β-CH_2_	4.01(dd); 2.87(dd),2.95(dd)	f
13	aspartate	α-CH; β-CH_2_	3.89(dd); 2.69(dd),2.80(dd)	f
14	butyrate	α-CH_2_; β-CH_2_; CH_3_	2.16(t); 1.56(m); 0.9(t)	f
15	choline	*N*-(CH_3_)_3_; α-CH_2_; β-CH_2_	3.20(s); 4.07(m); 3.52(m)	p/f
16	citrate	CH_2_(i); CH_2_(ii)	2.66(d); 2.54(d)	u/p
17	creatine	*N*-CH_3_; CH_2_	3.03(s); 3.92(s)	u/p
18	creatinine	*N*-CH_3_; CH_2_	3.03(s); 4.05(s)	u
19	cytidine	5-CH; 6-CH	6.10(d); 7.85(d)	f
20	cytosine	5-CH; 6-CH	5.98(d); 7.51(d)	f
21	D-3-hydroxybutyrate	α-CH_2_(i); α-CH_2_(ii); β-CH; CH_3_	2.41(dd); 2.31(dd); 4.16(m); 1.2(d)	p
22	Dimethylamine	2x CH_3_	2.72(s)	u
23	Glutamate	α-CH; β-CH_2_; γ-CH_2_	3.76(t); 2.07(m); 2.34(m)	f
24	Glutamine	α-CH; β-CH_2_; γ-CH_2_	3.78(t); 2.12(m); 2.43(m)	p/f
25	glycerophosphorylcholine	*N*-(CH_3_)_3_; α-CH_2_; β-CH_2_; α'-CH_2_; β'-CH; γ'-CH_2_	3.22(s); 4.32(t); 3.68(t); 3.61(dd); 3.90(m); 3.72(dd)	p
26	glyceryl of lipids	C***H***_2_OCOR	4.28(m)	p
27	Glycine	CH	3.55(s)	f
28	Hippurate	CH_2_; 2,6-CH; 3,5-CH; 4-CH	3.97(d); 7.84(d); 7.55(t); 7.64 (t)	u
29	Isoleucine	α-CH; β-CH; β-CH_3_; γ-CH_2 _(i); γ-CH_2_(ii); CH_3_	3.68(d); 1.98(m); 1.02(d);1.25(m),1.47(m); 0.94(t)	p/f
30	Lactate	α-CH; β-CH_3_	4.11(q);1.32(d)	u/p/f
31	Leucine	α-CH; β-CH_2_; γ-CH; 2x CH_3_	3.72(t); 1.74(m), 1.70(m); 0.96(t)	p/f
32	lipid fraction	C***H***_2_C = C	2.0(bs)	p
33	lipid fraction	C***H***_2_C = O	2.22(bs)	p
34	lipid fraction	C = CC***H***_2_C = C	2.75(bs)	p
35	lipid fraction	C***H***= C***H***	5.30(bs)	p
36	lipid fraction	C***H***_3_(CH_2_)_n_	0.83(bs)	p
37	lipid fraction	(C***H***_2_)_n_	1.22(bs)	p
38	lipid fraction	C***H***_3_CH_2_CH_2_C =	0.87(bs)	p
39	lipid fraction	C***H***_2_CH_2_CO	1.57(bs)	p
40	Lysine	α-CH; β-CH_2_; γ-CH_2_; δ-CH_2_; ε-CH_2_	3.78(t); 1.92(m); 1.72(m); 1.47(m); 3.03(t)	u/p/f
41	Methionine	α-CH; β-CH_2_; γ-CH_2_; *S*-CH_3_	3.87(t); 2.14(m); 2.63(t); 2.13(s)	f
42	*N*-acetylglycoprotein fraction	NHCOC***H***_3_	2.06(s)	u/f
43	Nicotinurate	2-CH; 6-CH; 4-CH; 5-CH; CH_2_	8.92(s); 8.70(d); 8.24(d); 7.60(dd); 3.99(s)	f
44	*p*-cresol glucuronide	2,6-CH; 3,5-CH; CH_3_	7.06(d); 7.23(d); 2.3(s)	u
45	Phenylacetylglycine	3,5-CH; 2,4,6-CH; Ar-CH_2_; *N*-CH_2_	7.43 (m); 7.37 (m); 3.75 (d); 3.68 (s)	u
46	Phenylalanine	2,6-CH; 3,5-CH; 4-CH; half Ar-CH_2_; half Ar-CH_2_; *N*-CH	7.33(d); 7.43(m); 7.36(m); 3.17(dd),3.30(dd); 3.99(dd)	f
47	Proline	α-CH; half β-CH_2_; half β-CH_2_; γ-CH_2_; δ-CH_2_	4.13(dd); 2.08(m), 2.37(m); 2.01(m); 3.38(m)	f
48	Propionate	CH_2_; CH_3_	2.19(m); 1.06(t)	f
49	Pyruvate	CH_3_	2.36(s)	u/p
50	*scyllo*-inositol	6x CH	3.33(s)	u/f
51	Succinate	2x CH_2_	2.41(s)	u/f
52	Taurine	*N*-CH_2_; SO_3_-CH_2_	3.43(t); 3.27(t)	u/f
53	Threonine	α-CH; β-CH; CH_3_	3.59(d); 4.27(m); 1.32(d)	f
54	Trimethylamine	3x CH_3_	2.89(s)	u/f
55	trimethylamine *N*-oxide	3x CH_3_	3.28(s)	u
56	Tyrosine	2,6-CH; 3,5-CH; α-CH; β-CH2	7.18(d); 6.88(d); 3.94(dd); 3.20(dd), 3.10(dd)	f
57	UN		4.11(bs); 3.75(m); 3.65(m)	p
58	Uracil	5-CH; 6-CH	5.80(d); 7.52(d)	f
59	Valine	α-CH; β-CH; γ-CH_3_; γ'-CH_3_	3.62(d); 2.28(m); 0.99(d); 1.04(d)	p/f
60	α-glucose	1-H; 2-H; 3-H; 4-H; 5-H; CH2(i); CH2(ii)	5.22(d); 3.54(dd); 3.71(t); 3.42(t); 3.83(ddd); 3.84(m); 3.76(m)	p/f
61	β-glucose	1-H; 2-H; 3-H; 4-H; 5-H; CH2(i); CH2(ii)	4.65(d); 3.24(dd); 3.48(t); 3.40(t); 3.47(ddd); 3.72(dd); 3.90(dd)	p/f

*s, singlet; d, doublet; t, triplet; m, multiplet; q, quadruplet; dd, double doublet; ddd, double double doublet; bs, broad signal.

An unsupervised PCA model was generated from the normalized NMR spectral data acquired from urine samples of both *S. mansoni*-infected and non-infected control mice at all time points using unit variance-scaled data. A total of three principal components (PCs) were used to construct the model and cumulatively explained 38.1% of the total variance (Figure 2C). Mice at an early stage of infection (≤ 41-day-old infection) showed similar trajectories to control samples, but from day 48 post-infection onwards, the infected mice showed different urinary trajectories along the first PC, forming a distinct cluster. The corresponding loadings plot indicated that PAG was the main metabolite influencing the separation between control and early-stage infected mice and mice at a later stage of infection.

**Figure 3:**
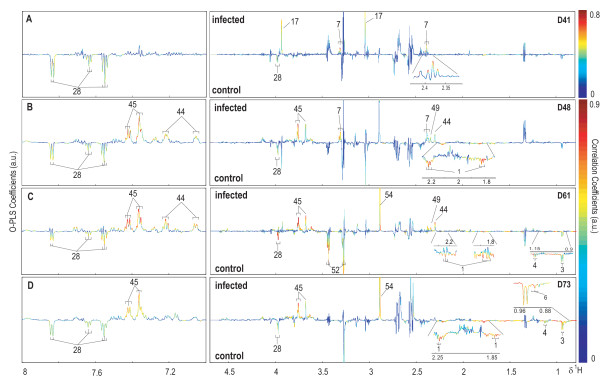
**O-PLS-DA coefficient plots derived from ^1^H NMR urinary spectra at day 41 (A), 48 (B), 61 (C) and 73 (D) (Q^2^*Y *= 0.58, 0.83, 0.94 and 0.86, respectively), showing the key metabolites discriminating between *S. mansoni*-infected mice and uninfected control mice**.

**Figure 4:**
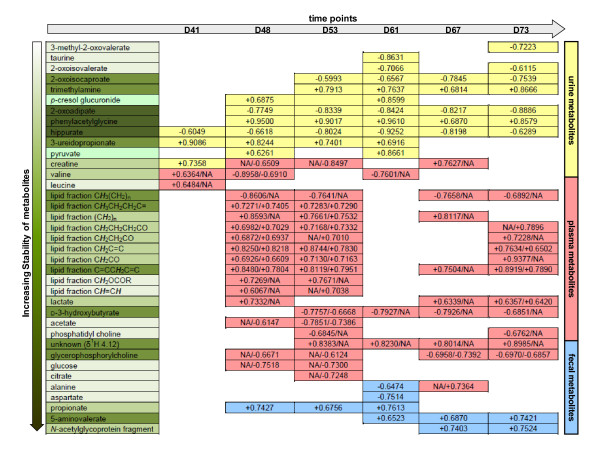
**Summary of *S. mansoni*-induced metabolic effects on biofluid composition of mice at six time points post-infection coded for biological matrix**. Colour keys are as follows: yellow, urine; red, plasma; blue, faecal water. Stability of metabolites, as candidate biomarkers of infection, is indicated by dark green through white corresponding to a progressive decrease in stability. Increasing stability of metabolites refers to the constancy of the metabolite as a significant biomarker for the discrimination of the infected mice from the controls during the entire course of the experiment. Numerical values are the coefficient covariance values for each metabolite and for plasma metabolites, 'A/B' format represents that value A is from models based on ^1^H standard plasma spectra, while value B is from models obtained from CPMG spectra. NA represents no significant correlation observed.

A series of pairwise O-PLS-DA models were constructed to model differentiation between *S. mansoni*-infected and non-infected mice at each sampling time point. No difference was observed between early-stage infected and non-infected control mice as shown in the PCA scores plot (Figure 2C). However, metabolic disturbances were identified in *S. mansoni*-infected mice from 41 days post-infection onwards. Selected O-PLS-DA coefficient plots (days 41, 48, 61 and 73 post-infection) are shown in Figure 3, and the dominant metabolite changes are summarized in Figure 4. Decreased levels of urinary hippurate and increased levels of creatine and 3-ureidopropionate were found in *S. mansoni*-infected mice at day 41 post-infection. Later in the experiment, hippurate levels continued to decrease, together with 2-oxoadipate, 2-oxoisovalerate, 2-oxoisocaproate and taurine. Whilst PAG concentrations were continuously higher in infected animals from day 48 post-infection onwards, *p*-cresol glucuronide, trimethylamine and pyruvate levels showed transient increases in urinary excretion in *S. mansoni*-infected mice on days 48 and 61. Over the entire course of the infection, hippurate, PAG, 2-oxoadipate and trimethylamine were found to be consistently different between the *S. mansoni*-infected and the non-infected control mice.

### Metabolic profiling and multivariate data analysis on mouse plasma

**Figure 5:**
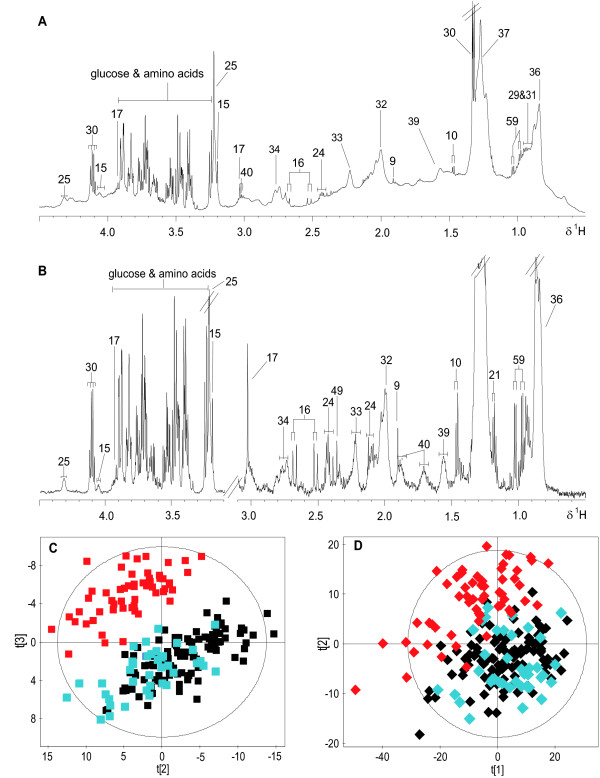
**Typical 1-D ^1^H NMR spectra of control mice acquired using standard (A) and CPMG (B) pulse sequences**. PCA scores plots derived from ^1^H standard (C) and CPMG (D) NMR spectral data (black, control; blue, *S. mansoni*-infected mice at the early stage of the infection (≤ 41 days); red, these mice at later stage of the infection (≥ 48 days)).

Metabolites identified in plasma spectra of non-infected control mice using either standard 1-D (Figure 5A) or CPMG (Figure 5B) pulse sequences are listed in Table 1.

An unsupervised pareto-scaled PCA model was calculated based on the normalized data from ^1^H standard (Figure 5C) and CPMG (Figure 5D) plasma spectra, derived from plasma samples of both control mice, and mice harbouring a pre-patent (days 13, 27, 34 and 41 post-infection) and a patent *S. mansoni *infection (days 48, 53, 57, 61, 67 and 73 post-infection). A three component model explained 73.7% and 48.1% of the total variance for the standard and CPMG data, respectively. Both of the scores plots (Figures 5C and 5D) represent a separation of the chronically *S. mansoni*-infected mice from the cluster of the non-infected control mice and those animals harbouring a pre-patent infection with a clearer differentiation in the standard than the CPMG spectral data. The corresponding loadings plots from the standard and CPMG models (data not shown) generated a similar ranking of discriminatory metabolites. The main observed infection discriminating features were higher levels of lactate and lower levels of *N*-(CH_3_)_3_-containing compounds, most likely a choline derivative. Further statistical correlation analysis showed that this signal was consistent with glycerophosphorylcholine and phosphatidylcholine, which were found to be higher in non-infected control mice compared with *S. mansoni*-infected mice.

In order to extract further biomarkers of *S. mansoni *infection, an O-PLS-DA method was employed on unit variance-scaled 1-D ^1^H standard and CPMG on days 13, 27, 34, 41, 48, 53, 61 and 73 post-infection and further models were calculated for diffusion-edited spectral data (only on two time points, days 48 and 53 post-infection). Changes in the plasma composition were observed at day 41 post-infection and at later time points. Strong models were obtained from standard ^1^H NMR spectra of plasma 48 days after an infection with Q^2^*Y *> 0.9. However, models derived from CPMG spectral data showed lower values of Q^2^*Y *compared with corresponding O-PLS-DA models of standard spectral data, with the exception of the 41 day post-infection sampling time point. Q^2^*Y *values of O-PLS-DA models of diffusion-edited spectra on days 48 and 53 were similar to those models of the standard spectra at the matching examination time points indicating that the infection induced a change in the plasma lipid composition.

**Figure 6:**
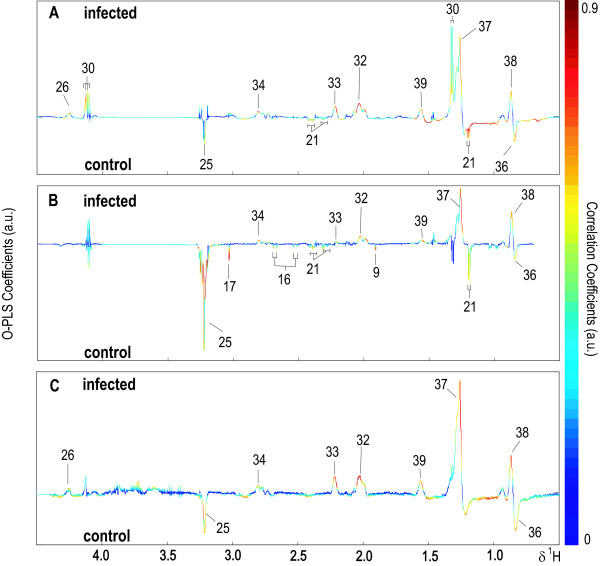
**O-PLS-DA coefficient plots derived from plasma spectral data acquired using standard (A), CPMG (B) and diffusion-edited (C) pulse sequences on 53 days post-infection showing discriminatory metabolites for infection**.

O-PLS-DA models derived from standard ^1^H NMR spectra, CPMG and diffusion-edited spectra of plasma collected from mice 53 days after infection with *S. mansoni *are depicted in Figure 6. A range of lipid fractions such as C***H***_3_CH_2_CH_2_C =, (C***H***_2_)_n_, C***H***_2_CH_2_CO, C***H***_2_C = C, C***H***_2_CO, C = CC***H***_2_C = C, C***H***_2_OCOR and -C***H***= C***H***- were present in higher intensities in the plasma of *S. mansoni*-infected mice, whilst lipid signals (e.g. C***H***_3_(CH_2_)_n _and CH_3_CH_2_C***H***_2_) and phosphatidylcholine, confirmed by STOCSY [18], were decreased. At day 53 post-infection, lower levels of several low molecular weight metabolites, including D-3-hydroxybutyrate, acetate and creatine, were observed in both standard and CPMG ^1^H NMR spectra of plasma samples derived from *S. mansoni*-infected mice, compared to uninfected control mice. Decreased levels of glucose and citrate were observed in the CPMG spectral data of the infected mice.

### Metabolic profiling and multivariate data analysis on faecal water from control and *S. mansoni*-infected mice

**Figure 7:**
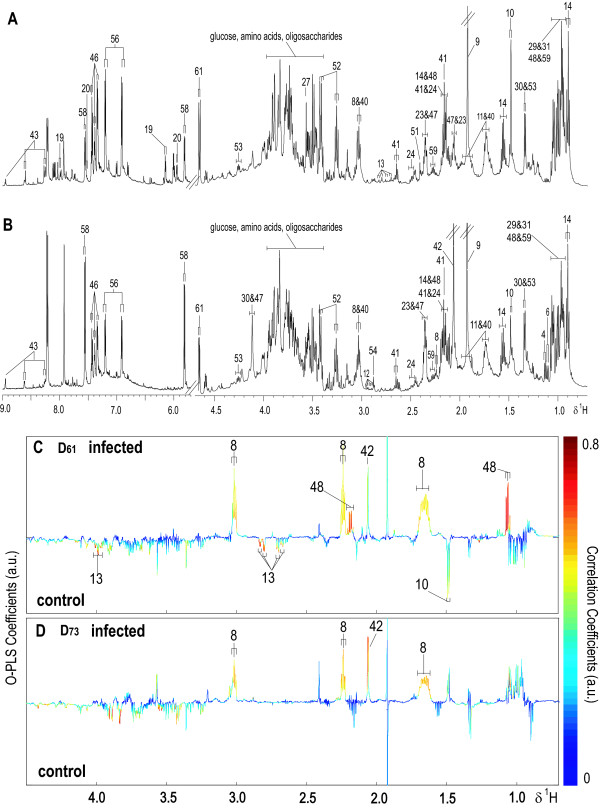
**Typical ^1^H NMR spectra of faecal extract samples obtained from an uninfected control (A) and *S. mansoni*-infected (B) mice at day 53 post-infection**. O-PLS-DA coefficient plots derived from ^1^H NMR faecal extract spectra at day 61 (C) and 73 (D) (Q^2^*Y *= 0.86 and 0.83, R^2^*X *= 49% and 66% of the total variance, respectively), reflecting the discrimination between the *S. mansoni*-infected and uninfected control mice.

Assignments for representative ^1^H NMR spectra of faecal water extracts obtained from a non-infected control mouse and a *S. mansoni*-infected mouse 53 days after the infection are shown in Figures 7A and 7B and include: amino acids (e.g. leucine, valine, alanine, isoleucine, aspartate, glutamine, glutamate, lysine, arginine, glycine, methionine, phenylalanine and tyrosine), short chain fatty acids (SCFAs; e.g. butyrate, propionate and acetate), uracil, 5-aminovalerate, nicotinurate, succinate and glucose.

Unsupervised PCA analysis did not yield a clear separation between the *S. mansoni*-infected and non-infected control mice due to large variance in the metabolic profiles of faecal extract samples. Significant differentiation in faecal profiles from infected and non-infected mice was achieved using pairwise O-PLS-DA models constructed for each time point based on unit variance-scaled faecal extract spectral data using one PLS predictive and two orthogonal components. The coefficient plots (Figures 7C and 7D) showed the disturbance of a number of metabolites, such as increased propionate, 5-aminovalerate and *N*-acetyl glycoprotein signal in *S. mansoni*-infected mice, which were apparent from day 48.

### Exploration of the correlation between selected urinary metabolites, discriminatory for infection, and faecal water profiles

**Figure 8:**
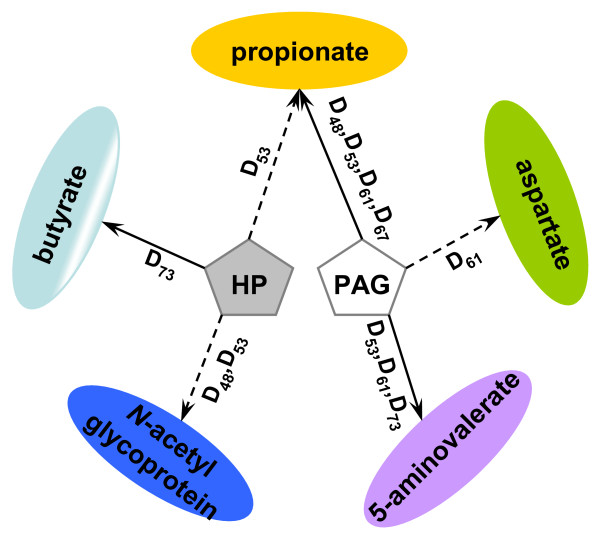
**Schematic illustration of correlations between faecal metabolites (e.g. propionate, aspartate, 5-aminovalerate, *N*-acetylglycoprotein and butyrate) and urinary hippurate (HP) and phenylacetylglycine (PAG) levels**. A solid line represents positive correlation between two metabolites, whilst a dashed line represents negative correlation. D_x _indicates the data points at which the correlation was observed.

Urinary levels of hippurate and PAG were found to be consistently disturbed in mice infected with *S. mansoni *from days 41 post-infection onwards. Since both these metabolites derive from gut microbial-host co-metabolism, an O-PLS regression analysis was applied to the spectral data of faecal extracts against the relative concentration of each metabolite in order to characterize the link between these two urinary metabolites and faecal extract profiles. O-PLS regression of urinary hippurate against the faecal extract profiles generated robust models at the following sampling time points: 48 days post-infection (Q^2^*Y *= 0.58, R^2^*X *= 80%) with one predictive component and two orthogonal components, 53 days post-infection (Q^2^*Y *= 0.57, R^2^*X *= 39%) and 73 days post-infection (Q^2^*Y *= 0.58, R^2^*X *= 29%) with one predictive component and one orthogonal component. *N*-acetyl glycoprotein fragments and propionate were negatively correlated with the urinary hippurate levels, whilst butyrate was found to be positively correlated with hippurate at day 73 post-infection (Figure 8). No correlation between hippurate levels and faecal metabolites were observed at other sampling time points. O-PLS regression of urinary PAG against the faecal extract profiles calculated using one aligned and two orthogonal components generated stronger models than hippurate at all examination time points: Q^2^*Y *= 0.56 and R^2^*X *= 63.3% at day 48 post-infection; Q^2^*Y *= 0.80 and R^2^*X *= 56.4% at day 53 post-infection; Q^2^*Y *= 0.75 and R^2^*X *= 47% at day 61 post-infection; Q^2^*Y *= 0.55 and R^2^*X *= 53.6% at day 67 post-infection; and Q^2^*Y *= 0.76 and R^2^*X *= 64.2% at day 73 post-infection. Propionate and 5-aminovalerate were found to be highly correlated with the urinary PAG levels, whilst aspartate was negatively associated to PAG at day 61 post-infection (Figure 8).

An O-PLS regression was also performed on biofluid spectral data obtained at the last time point against worm burden, but no significant correlation was observed.

## Discussion

Dynamic metabolic signatures of mice experimentally infected with *S. mansoni *were generated for urine, plasma and faecal water. In preceding metabolic profiling studies we selected 49 and 56 days post-infection as sampling time points of overt infection, since previous studies [19] and in-house investigations revealed that oviposition occurred, on average, 34-35 days after mice were infected with *S. mansoni*. However, literature suggests that there is considerable heterogeneity in the timing of oviposition in murine models depending on strain of host and parasite [20]. We therefore extended the temporal range of sampling to give opportunity for early detection of response to infection and to generate a more holistic picture of the emerging infection. In the current study we detected a significant metabolic response at 41 days post-infection in both the urine and the plasma profiles. The key discriminatory urinary metabolites at this earlier time point were decreased urinary hippurate and increased levels of creatine and 3-ureidopropionate, whilst the response in plasma at this time point was manifested by increased valine and leucine. As the infection developed the global urinary response observed here was consistent with previous publications that showed a strong metabolic response at 49 days post-infection consisting of modulation of oxoacids, methylamines, tricarboxylic acid intermediates and gut microbial-mammalian co-metabolites. The sampling strategy allowed the construction of metabolic trajectories for each biofluid (Figure 4) and gave an indication of which metabolic changes persisted throughout the duration of the infection. Urinary hippurate, PAG and 2-oxoadipate were the most stable predictors of infection over the experimental period, whereas modulated levels of 2-oxoisocaproate and trimethylamine in urine, lipid components and D-3-hydroxybutyrate in plasma and faecal 5-aminovalerate were robust at the later time points. A number of metabolites demonstrated a transient response to infection including urinary taurine and pyruvate, plasma acetate and lactate, faecal propionate and *N*-acetylglycoprotein fragments. These transient alterations may reflect particular time-dependent responses to *S. mansoni *infection or may simply be more variable in concentration and therefore less robust as biomarkers.

### *S. mansoni*-induced anaemia in mice

The dramatic decrease in the PCV of mice 48 days after experimental infection with *S. mansoni *indicates that animals suffered from anaemia. There are several possible mechanisms that might have caused anaemia. For example, iron deficiency due to the blood loss in faeces, hepatosplenomegaly resulting in erythrocyte sequestration and inflammation-related anaemia [21]. These mechanisms are supported by our current observations, including observation of blood in faeces from some of the infected mice at several time points from day 48 post-infection onwards, spleen enlargement in all the *S. mansoni*-infected mice on dissection (histological evidence was reported in a previous study [14]) and elevated levels of several cytokines, such as interferon-γ (IFN-γ), tumor necrosis factor-α (TNF-α), interleukin-4 (IL-4), IL-5 and IL-12 in plasma in response to the infection [22]. Although *S. mansoni*-induced anaemia could involve multiple mechanisms, including the consumption of red blood cells by adult schistosomes, we speculate that splenomegaly and inflammation play key roles, leading to host anaemia, since blood loss in faeces were only found in some of the infected mice.

### *S. mansoni*-induced disturbance of phospholipids metabolism

Phospholipids are believed to act as important mediators in immunological modification and signal transduction [23]. Schistosomes are incapable of synthesizing fatty acids *de novo*, thus they must absorb and utilize exogenous lipids from the host's blood and incorporate them into their membrane structures in order to evade the host immune response [24]. The decreased level of phospholipids such as phosphatidylcholine in plasma from the *S. mansoni*-infected mice could be due to absorption of phosphatidylcholine by the parasite. Alternatively, the parasite could provide free choline by its breakdown to diglycerides synthesized within schistosomes, which may also explain the lower levels of glycerophosphorylcholine in plasma of infected mice. This finding is in agreement with Rumjanek and Simpson's previous research on lipid utilization and incorporation by *S. mansoni *worm *in vitro *[25].

### *S. mansoni*-induced energy metabolism disturbance

Several of the metabolic changes observed in the *S. mansoni*-infected mice, particularly the finding of markedly lower concentrations of glucose at certain time points or increased lactate in plasma from infected mice suggest an alteration in energy metabolism. Pairs of adult schistosomes live in the mesenteric veins, where they depend on glucose uptake from the host to provide energy for their survival and egg production [26] and thus the reduction in plasma glucose may simply reflect increased utilization by the parasite. In our previous study with a focus on metabolic alterations in tissue samples obtained from *S. mansoni*-infected mice 73 days post-infection, we noted high levels of pyruvate and low levels of glucose and glycogen in livers of *S. mansoni*-infected mice [14]. Taken together with the increased plasma lactate, and the observation that activities of glycolysis-involved pyruvate kinase and phosphofructokinase in the liver were reported to be greater in *S. mansoni*-infected mice [27], this would suggest an infection-induced stimulation of glycolysis.

Urinary levels of 2-oxoisovalerate, 2-oxoisocaproate and 2-oxoadipate were decreased in the current and a previous study [9]. These three metabolites are known to originate from valine, leucine and α-aminoadipate, and of these valine and leucine were found to be accumulated in the liver from *S. mansoni*-infected mice [14]. The increase of these metabolites suggests a disturbance of amino acids metabolism, and is likely to reflect liver dysfunction.

### *S. mansoni*-induced microbial disturbance

Previous studies profiling the urinary response of rodent and human hosts to *S. mansoni *infection have reported gross disturbance of metabolites associated with either gut microbial or microbial mammalian co-metabolism such as increased PAG and decreased hippurate [5]. By profiling of time-dependent changes in the chemical composition of faecal extracts and *via *direct correlation of the two strongest infection-related urinary indices of gut microbial metabolism (i.e. PAG and hippurate), we add further evidence to the hypothesis that *S. mansoni *infection either directly or indirectly modulates host gut microbial activity. As with previous studies, levels of PAG, hippurate, trimethylamine and *p*-cresol glucuronide were changed in the urinary profiles following infection. Both hippurate and PAG are absent in the urine of germ-free rats and are observed only after 24-48 hours and 20 days, respectively, following exposure of these animals to the laboratory environment indicating their association with microbial metabolism [28]. Hippurate, formed by conjugation of glycine with benzoate in the liver mitochondria [29], can be derived from dietary sources but since the infected and non-infected mice were supplied with the same diet, and there was no difference in the mean body weights of these two groups, this would rather support the alteration of microbial composition or activity. *p*-Cresol is a metabolite of protein degradation and subsequent to its synthesis from tyrosine it undergoes hepatic glucuronidation or sulfation prior to urinary excretion [30]. Its production is subject to intestinal environment such as composition of microbiota, food intake (protein intake, particularly tyrosine) and pH of the intestinal tract [31]. *Clostridium difficile *and *Lactobacillus *strains are known to produce *p*-cresol by decarboxylation of *p*-hydroxyphenylacetate [32,33]. Thus, elevated urinary levels of *p*-cresol glucuronide observed in infected mice may indicate that *S. mansoni *could activate those *p*-cresol-producing bacteria strains or somehow disrupt the microbial ecology favouring the presence of activity of *p*-cresol producing bacteria.

Trimethylamine (TMA) can be derived from dietary choline *via *microbial processing and eventually excreted into the urine after being absorbed by microvillae and circulated to the kidney *via *the bloodstream [34].

In addition to the urinary microbial metabolites, several faecal metabolites also pointed to altered microbial metabolism. 5-Aminovalerate and SCFAs, including propionate, were increased in faecal extract samples from infected mice. In particular, 5-aminovalerate was consistently increased and stable changes during the last three time points. *Clostridium sticklandii *and *C. valericum*, gram-positive anaerobic bacteria belonging to the proteolytic clostridia, can produce 5-aminovalerate from proline [32,35]. SCFAs originate from bacterial fermentation of non-digestible starch or oligosaccharides and the production of SCFA relies on bacteria species and intestinal environment (e.g. pH) [36]. Correlations between urinary and faecal gut microbial metabolites, such as the positive correlation of urinary hippurate with faecal butyrate or the negative association between urinary PAG and faecal propionate, further suggests a coordinated systems response at the level of the interaction between parasite, host and enteric microbiota.

**Figure 9:**
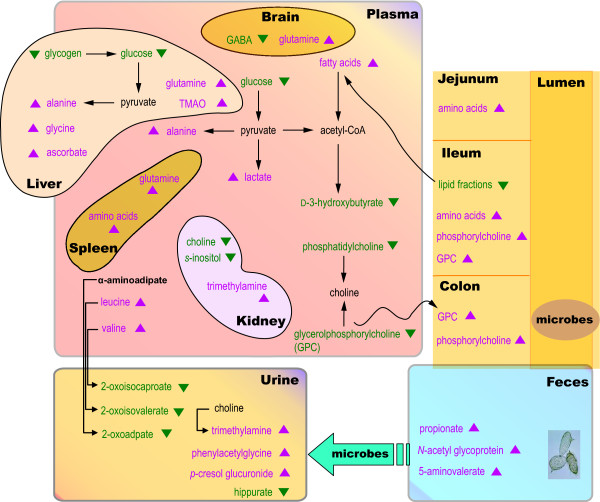
**Schematic illustration of systematic changes observed in biofluids from *S. mansoni*-infected mice and their interrelationships with observations from tissue analysis at the point of established, chronic infection at day 73 post inoculation adapted from reference **[14]**)**. Key: TCA, tricarboxylic acid.

The global response derived from the correlation of metabolic profiles obtained from urine, plasma and faeces points to both local and systemic effects of *S. mansoni *infection (Figure 9), similar to observations on other parasitic infections such as *Echinostoma caproni *[37]. Dominant themes of *S. mansoni *infection include altered energy metabolism, gut microbial activity and immunological response. *S. mansoni *eggs play a major role in triggering a variety of host immune responses by secreting glycoproteins, glycolipid antigens and unconjugated oligosaccharides [38-40]. Thus the markedly higher amount of *N*-acetyl glycoprotein fragments in mice with 67- and 73-day-old infection, may reflect a response to the eggs rather than the worms since acute phase glycoproteins have been associated with inflammation [41].

## Conclusion

The systems metabolic change in the mouse due to *S. mansoni *infection reported here extends previous knowledge on the host-parasite interaction in the following aspects: (i) changes in urine were observed as early as 41 days post-infection; (ii) plasma and faecal extract profiles provide complementary information to urine profiles on the systemic perturbation induced by *S. mansoni *infection and in particular develop the evidence-base for parasite-induced disruption of gut microbial functionality. This NMR-based metabolic profiling approach proved useful and robust for monitoring biological changes in biofluids at the molecular level. Compared with other host-parasite models studied before, the metabolic interactions between host and parasite shared some similarities, however, each parasite examined also demonstrated a unique metabolic signature, which is promising in terms of developing a robust diagnostic tool for both individual and population levels.

## Competing interests

The authors declare that they have no competing interests.

## Authors' contributions

JVL, JS, YW, JK, JU and EH designed research; JVL, JS and JK performed animal experiments; JVL. performed NMR analyses and bioinformatic analyses; JVL, JS, YW JK, JU and EH interpreted data and wrote the paper. All authors read and approved the final submitted and revised versions of the manuscript.
